# Contributions to a Knowledge of the Influence of Employments upon Health

**Published:** 1845-10

**Authors:** 


### Art. XI.

1. *Contributions to a Knowledge of the Influence of Employments upon Health.* 2. *Further Contributions to a Knowledge of the Influence of Employments upon Health.* 3. *A Third Contribution to a Knowledge of the Influence of Employments upon Health.* 4. *Health of Towns Commission. Minutes of Evidence given by William Augustus Guy,* M.B. *on the Influence of Employments upon Health.* By William Augustus Guy, M.B. Cantab., Professor of Forensic Medicine, King's College, and Physician to King's College Hospital.

The recent numbers of the 'Journal of the Statistical Society' have contained several valuable contributions to vital statistics. The papers of Mr. Chadwick and Mr. Neison have already been noticed on a former occasion;* we propose in this place to give an abstract of Dr. Guy's essays, on the important subject of the influence of employments upon health, with which we shall combine what appears to be their complement, the Minutes of the Evidence on the same subject given before the Health Commission, and already cursorily noticed in our review of their recently published volume.

The essays before us, contain no less than forty-five elaborate tables, besides a sheet of illustrative causes; they may be fairly expected, therefore, to display some new facts, and to suggest some considerations of

* British and Foreign Medical Review, vol. XVIII, p. 197.

interest to the medical man as well as to the public. We shall endeavour to exhibit the principal results without the aid of tables, and we shall, for perspicuity's sake, adopt an arrangement differing from that which the author has employed.

The materials for the essays have been obtained from two distinct sources; the mortuary registers of the metropolis for 1839, and the out-patient books of the King's College Hospital. The evidence laid before the Health Commission is based chiefly upon a personal inspection of the workshops of the metropolis, especially of the printing-offices. We shall state the results derived from each of these sources in turn, beginning with those from the mortuary registers.

The average age at death of different classes of the community, is a point of much interest which has already been amply illustrated by Mr. Chadwick in his valuable Sanitary Reports. From one of Dr. Guy's tables, contrasting the three classes of gentlemen (including professional men), tradesmen, and artisans (including the entire labouring class), it appears that the average age attained by gentry and professional men is little short of 59 years, while the tradesmen live only 49, and the labouring class little more than 48. These averages are formed from the deaths occurring at 15 years of age and upwards. The greatest age attained by members of the first class was 98, it was 97 in the case of tradesmen, and 101 in the case of the labouring class.

The great difference between the average age of the first class and of the last two classes, and the very slight difference between that of tradesmen and the labouring class, are equally worthy of observation. As all deaths taking place in workhouses among persons whose occupations are not stated are omitted, and as it is probable that the greater number of such persons belong to the labouring class, it is obvious that the average is merely an approximation. It is also below rather than above the true average, as the mean age of adults dying in workhouses is as high as 60 years. On the other hand, it must be borne in mind that a certain proportion of the class of tradesmen, especially the petty tradesmen, originally belonged to the labouring population, and had been for a part of their lives submitted to the influences, whether injurious or otherwise, to which the labouring population is exposed. If allowance were made for both these disturbing causes, it is probable that the average result would not differ materially from that just stated: the tradesmen and the entire adult labouring population would be found to be in nearly the same sanitary condition, while the lives of the adults of the more favoured classes, taken one with another, would be lengthened by about a fifth of the mean duration of life of the adult trading and labouring population.

The influence of different classes and kinds of employment, and of one baneful habit, that of intemperance, is examined in a series of elaborate tables. The effect of in-door as contrasted with out-door occupation, is the first object of inquiry. It appears that the average age at death is lower in the first class than in the second, being in the one case 47 years and in the other 49 years; that the deaths under 30 in the one class form nearly 22 per cent. of the total deaths, and in the other only 17 per cent., and that under 40 years of age the per centage proportions are respectively 39 and 34 per cent. In-door employments, then, are evidently much more injurious to health than out-door occupations; all in-door employments, however, are not unhealthy in an equal degree. This is shown in a table in which the several in-door employments are grouped according to the amount of exertion which they require, the first group consisting of those requiring little exertion, the third of those demanding great exertion, while a second group combines all those requiring an intermediate degree of exertion. In the first group the mean age of death is, in round numbers, 47 years, in the second and third 48 years; but those employments requiring great exertion exhibit a somewhat lower average age at death. The greatest age attained (101 years) is in the second class, the lowest, less than 90, in the class requiring great exertion. The maxima for the three classes in the order in which they have been named is 98, 101, and less than 90. The deaths under 30 in the three classes are inversely as the amount of exertion; being in the sedentary class 24 per cent., in those using great exertion 19 per cent., and in those using still greater exertion 18 per cent. Under 40, the deaths for the three classes respectively are 40, 37, and 34 per cent. The general result which may be deduced from this table then is, that in men employed in-doors the average age of death is lower as the amount of exertion is less; but that the greatest age is attained by men using moderate exertion, while those using little exertion attain a greater age than those using great exertion. To put this statement into its shortest form—sedentary habits are unfavorable to health and long life in the many, but favorable to longevity in the few; on the other hand, strong exertion is more favorable to health but less favorable to longevity. A moderate degree of exertion is most favorable to the attainment of old age. It would appear, too, that the habits of the labouring class, though on the whole less favorable to the health of the entire class than the condition of the gentry or tradesmen, is more favorable to longevity. The most remarkable examples of longevity have also occurred in that class.

The injurious effects of habits of intemperance are strikingly displayed in a table contrasting the age at death of men exposed to the temptation of drinking, with that of men following similar employments, but not unusually open to such temptations. The most accurate and unexceptionable comparison is that between the drayman and the labourer. The average age of the former is 43, that of the latter 47½ years. The greatest age attained by any of the former class is less than 90 years, while 98 is the maximum in the case of the latter. In the interval from 30 to 40, as many as 39 per cent. of the draymen die, but only 18½ per cent. of the labourers. Though no additional proofs of the fatal effects of intemperance were required, it may be well to put on record this strong confirmation of a familiar fact.

Such are the *most interesting* results of the tables founded upon the ages at death, irrespective of the cause of death. Another and more lengthened series of tables illustrates the influence of similar conditions and modes of life on the production of pulmonary consumption, the principal fatal disease of the adult.

The relative liability to consumption of the gentry, tradesmen, and labouring men is shown in a table which presents at a glance the per centage proportion of deaths at different ages, the average age at death, and the ratio which deaths from pulmonary consumption bear to those by all other diseases. The per centage proportion of deaths occurring under 30 in the three classes, in the order in which they have been just named, viz. the gentry, the tradesmen, and the labouring men, is $29\frac{1}{2}$, 33, and 31. Under 40 the numbers are $56\frac{1}{4}$, 60, and 57. The average ages are 39, 38, and $38\frac{1}{3}$; and the ratios of deaths by consumption to those by all other diseases are respectively 1 to 5, 1 to $2\frac{1}{2}$, and 1 to $2\frac{1}{4}$. The two classes of tradesmen and labouring men, therefore, are nearly twice as liable to attacks of pulmonary consumption as the class of gentry, and the difference between the first two classes is very trifling. In respect of the average age at death, and the per centage proportion of deaths under 30 and 40 years of age a curious circumstance is observable, namely, that the tradesmen who die of consumption die at an earlier age than the gentry or the labouring class; the cause of which will presently appear. The comparative liability to consumption of the in-door and the out-door labourer is shown in a table corresponding to the table of deaths from all causes. The per centage proportion of deaths from consumption is much higher in the first than in the second class, the numbers being—in-door $37\frac{1}{2}$, out-door 25: under 40 the numbers are—in-door 61, out-door 53. The ratios of death from consumption to those by all other diseases are respectively 1 to 1·98, and 1 to 2·56, or in round numbers 1 to 2 and 1 to $2\frac{1}{2}$.

The deaths from consumption among men working in-doors, are greatly influenced by the amount of exertion. Thus while 44 per cent. of men following sedentary occupations die of consumption before 30 years of age, 31 or 32 only of those using greater exertion fall victims to that disease before the same age. The numbers dying before 40 are respectively 66 per cent., and 54 or 55.

We have already drawn attention to the fact that the tradesmen fall victims to consumption at an earlier age than the gentry, or the labouring class. It appears from another table that they occupy a middle place between in-door and out-door labourers, and between men following sedentary occupations within doors, and those using greater exertion in their in-door employments. The following comparisons will make this more clear: Deaths under 30:—in-door labourers $37\frac{1}{2}$, tradesmen 33, out-door labourers 25; in-door sedentary 44, tradesmen 33, in-door requiring great exertion $31\frac{1}{2}$. By thus dividing the whole labouring class into two groups, the cause of the earlier liability of the tradesmen to consumption becomes obvious. The entire labouring class is more favorably circumstanced in this respect, because it includes a considerable number who either work out of doors, or following their employments in-doors, make use of strong exertion. The tradesman is confined during the entire day to his shop, and loses the benefit of the open air and exercise which fall to the lot of the out-door labourer; he uses little exertion within doors, and consequently suffers in the same way as those who follow sedentary occupations within doors. His sanitary condition is little more favorable than that of the tailor or compositor. The results obtained from the mortuary registers are borne out by a series of tables exhibiting the ratio of consumptive cases to those of all other diseases, and the age at which consumption has made its attack, among the out-patients of the King's College Hospital. The ratio among men working in-doors is 1 to 3·81, among those working out of doors 1 to 4·13. For men working in-doors and using different degrees of exertion the ratios are: sedentary 1 to 3·08, more exertion 1 to 4·44, great exertion 1 to 5·06. The numbers attacked under 30 are as follows: in-door 51 per cent., out-door 36 per cent.; sedentary 51 per cent., more exertion 53 per cent., great exertion 49 per cent. Under 40 the numbers are still more uniform. They are as follows: in-door 83 per cent., out-door 62 per cent., sedentary 81 per cent, more exertion 80 per cent., great exertion 67 per cent. These results, in the case of in-door and out-door occupations, are fully confirmed by tables comparing the cases of consumption occurring among females following these two kinds of employment.

The unhealthiness of occupations requiring little exertion, as compared with those demanding greater efforts, is exemplified in the case of the compositor as compared with the pressman. Both work in rooms similarly warmed, and equally ill ventilated, and both breathe the same atmosphere, but the former uses little exertion, and the latter much. The ratio of consumptive cases to those of all other diseases among compositors is 1 to 3·47, and among pressmen 1 to 5·12.

It is but natural to suppose that employments which are unfavorable to health should number a greater proportion of young persons than more wholesome occupations. This accordingly is found to be the case, and the degree of difference is shown in a series of tables. This disproportion in the age of men following more or less healthy occupations is used by Dr. Guy as a concurrent probability in support of the conclusions drawn from the tables of diseases and deaths. It is obvious, however, that this test is a fallacious one, for the ages may be influenced by other causes, as by an increased demand for young persons in one class of occupation as compared with the other. Take, for instance, the compositors and pressmen. It appears from Dr. Guy's tables, that the average age of compositors is less than that of pressmen. Does this necessarily result from the occupation of the compositor being more fatal to life than that of the pressman? There is only one way in which this question can be satisfactorily answered, and that is by ascertaining the existing age of several men following both occupations and beginning them at the same age, and then comparing the average age of the two classes. This comparison Dr. Guy has instituted on a limited scale, and has laid the result before the Health Commission; and it appears that when compositors who began to work as such at 14, 15, and 16 years of age, are compared with pressmen beginning their work at the same ages, the mean age of the one is 28 years, that of the other 34 years, showing a loss on the part of the compositor of 6 years. A similar result is obtained by comparing the two classes beginning their employment at the ages of 14, of 15, and of 16 years—the difference in favour of the pressmen being 3, 10, and 8 years respectively. The greatest age attained in the case of the pressmen is 60, and in the case of the compositors 72. This corresponds with what has been already remarked, that sedentary occupations are favorable to longevity, while those requiring stronger exertion yield, so to speak, a better average result.

There are many other facts established in these essays, and illustrated by tables which our space will not allow us to notice at any length; we shall, therefore, content ourselves with extracting from the second essay the author's summary, making such slight additions from the third essay as will render that summary complete. A comparison of in-door with out-door occupations leads to the following results:

1. The ratio of cases of pulmonary consumption to those of other diseases is somewhat higher in persons following in-door occupations, than in those working in the open air; and this rule applies to both sexes. It also holds good with regard to the deaths from consumption as compared to those from all other diseases.

2. Pulmonary consumption occurs at an earlier age in men following in-door occupations than in those following out-door occupations. The same rule holds good with regard to the deaths from consumption.

3. The probable excess of cases of pulmonary consumption in men following in-door occupations (for a higher ratio of consumptive cases in those employments is merely a strong presumption in favour of such excess,) and the earlier age at which consumption makes its attack, would naturally tend to fill this class of employments with a greater number of young men, as well as to occasion a higher mortality at the early periods of life, and a lower average age of death. Accordingly, among those employed within doors, there is an excess of young persons, a higher mortality in early life, and a lower average age of death. The greatest age, as it happens, is the same in the two classes.

The classification of in-door employments, according to the amount of exertion which they require, leads to the following results:

1. The ratio of cases of pulmonary consumption to those of all other diseases is highest when the amount of exertion is least, and lowest when it is greatest; and the intermediate degree of exertion presents an intermediate ratio. The same statement holds good in respect of the deaths from consumption compared with those from other diseases.

2. The age at which consumption makes its attack, and at which it proves fatal, is earlier in employments requiring little exertion than in those requiring more exertion, and in those requiring moderate exertion than in those demanding great effort.

3. The per centage proportions of men under 40 years of age following these three classes of employment is in strict accordance with the ratio of consumptive cases, and the ages at which consumption makes its attack and proves fatal; these proportions following the exact order of the degree of exertion.

4. The age of death, and the age of death from consumption also follows the same order, the per centage proportion of deaths under 40 being highest when there is least exertion, lowest when there is greatest, and intermediate when there is an intermediate degree of exertion.

5. The average age of death, also, is lowest when there is least exertion; but highest when the exertion is intermediate between the two extremes. The somewhat lower average obtained in the case of employments requiring great exertion, appears to depend on an excessive mortality under 20 years of age. The greatest age also occurs in occupations requiring a medium degree of exertion, the least maximum in those demanding the greatest effort.

6. In the class of in-door occupations, with varied exercise, (a class including the footman, waiter, &c.,) the ratio of cases of pulmonary consumption ranks next to that of the sedentary occupations, and the per centage proportion of consumptive cases occurring before 40, the per centage proportion of men so employed under that age, and the per centage proportion of deaths are higher than in any of the other classes, while the average age of death is lower. This class, however, stands alone, inasmuch as young men are in greater request, and old men comparatively little wanted.

7. The class of out-door occupations requiring moderate exertion presents a higher per centage proportion of deaths under 40, and a corresponding excess of young men; but the ratio of consumptive cases and the per centage proportion of such cases occurring under 40, are lower than in the class requiring greater exertion. This apparent anomaly may probably be explained by the fact that the attack of consumption is postponed till a later age in men following out-door employments, than in those working within doors.

8. The maximum age in the case of men following the more laborious out-door employment is lower by one year than in those using less-exertion, and in the latter there is a considerable excess of aged men.

9. Sedentary employments, and those requiring little exertion, are more unfavorable to adult and middle age, but more favorable to old age, than those requiring greater effort; on the other hand, employments requiring great exertion are unfavorable to youth and longevity, but favorable to middle age. Employments requiring little exertion prove fatal by inducing an excess of cases of pulmonary consumption, those requiring great exertion by occasioning other diseases of the air-passages and lungs, towards the commencement of old age.

The following observations apply to certain occupations examined separately, and to the effects of intemperance:

1. The exposure to a high temperature does not appear to exercise any injurious influence upon health during the early periods of life; but, in common with employments requiring a great amount of exertion, it is unfavorable to longevity.

2. The inhalation of dust does not appear to be attended with the extremely injurious consequences which the high ratio of cases of pulmonary consumption would lead us to expect; but when compared with the aggregate of other out-door occupations, the employment of the mason is found to be, in some degree, less favorable to health and longevity.

3. Habits of intemperance appear to exercise a most injurious influence upon health; for men peculiarly exposed to the temptation of drinking present a high ratio of cases of consumption, a high per centage proportion of such cases occurring under forty, an excess of young men, an excess of deaths under forty, and especially between thirty and forty years of age, and a low average and maximum age.

Lastly. A comparison of the ages at death of gentlemen, (including professional men,) tradesmen, and artisans, issues in displaying the great advantage which the first class possesses over the other two, and the comparatively small difference which exists between the tradesman and the artisan. The average age of death of the first class exceeds that of the other two by 10 years, while the average age at death of the tradesman exceeds that of the artisan only by a small fraction of a year.

Such are the chief results to which Dr. Guy's tables and reasonings lead him. The point on which he lays the greatest stress is the tendency of certain classes of employment to promote pulmonary consumption, the liability to this disease being taken as a sort of test and measure of the influence of employments upon health. Having confirmed the conclusions of other observers as to the tendency of sedentary occupations to promote pulmonary consumption, he endeavours to ascertain the cause of their unhealthiness by comparing persons using different degrees of exertion, but breathing the same air, as, for instance, the compositors and the pressmen, and he shows satisfactorily that the first class suffer more than the last. This may be explained in one of two ways—either by a want of proper exercise alone, such inertness being in itself injurious, or by the circumstance that comparative inaction increases the injurious effects attributed to hot and foul air. This latter alternative is the one towards which Dr. Guy evidently leans, and he supports his view by instancing the good health and long life enjoyed by literary men leading sedentary lives, but, unlike the artisans, breathing a pure air.

In the evidence given before the Health Commission, some very striking facts are stated illustrative of the injurious effect of a heated and foul atmosphere. Three several comparisons of a very precise kind, in which the state of health of men having different proportions of air to breathe was inquired into, agree in displaying the sad effects produced by the total neglect of ventilation. The most remarkable comparison is exhibited in a table which contrasts three groups of men having respectively less than 500, between 500 and 600, and more than 600 cubic feet of air to breathe. Among the first group $12\frac{1}{2}$ per cent. had suffered from hemoptysis, and the same number from frequent attacks of catarrh; of the second group only $4\frac{1}{3}$ per cent. had spit blood, and $3\frac{1}{2}$ per cent. were subject to cold; while of those having more than 600 cubic feet of air to breathe, less than 4 per cent. had had hæmoptysis, and less than 2 per cent. complained of a liability to catarrh. The subjects of the comparisons were letter-press printers.

Before we dismiss Dr. Guy's essays we must notice an interesting calculation or estimate which he has formed of the number of deaths from pulmonary consumption occurring in England, and of the probable waste of life which occurs in the metropolis and in the large towns.

The deaths entered yearly in the returns of the Registrar-general under the head of consumption, amount to little less than 60,000. Of these nearly a half occur at ages at which pulmonary consumption is acknowledgedly of rare occurrence. Thus, of 7282 deaths entered as consumption in the mortuary registers of the metropolis on the average of the two years 1840 and 1841, as many as 1560 occurred before fifteen years of age, and 374 after sixty years of age, leaving for the interval from fifteen to sixty only 5344 deaths. Many of the deaths under fifteen occurred at very early ages, and many of those after sixty at periods of life at which true pulmonary consumption is known to be extremely rare. Tubercular deposits in the lungs, it is true, are often met with in the bodies of children dying of other diseases, but death from pulmonary consumption among children is known to be comparatively rare. In endeavouring to correct these evidently erroneous returns, Dr. Guy starts with the assumption that the number of deaths entered as consumption between the ages of fifteen and sixty, are a near approximation to the true number, and then proceeds to calculate the number which may be supposed to occur before fifteen and after sixty, by means of the deaths occurring at those ages in the London Hospitals during the year 1840. It is obvious that this mode of estimating the number of deaths from consumption is merely a very general approximation, which may require correction; but it is doubtless much nearer to the truth than the numbers given in the Reports of the Registrar-general. Adopting this mode of calculation, Dr. Guy reduces the number of deaths occurring annually in the metropolis from pulmonary consumption to 5560, and by means of a calculation, in which we do not think it necessary to follow him, he estimates the total annual mortality for England and Wales at very nearly 36,000. He estimates the mortality from consumption in the metropolis at one eighth of the deaths at all ages, and somewhat less than a fourth of the deaths occurring above fifteen years of age; and for England and Wales at less than one ninth of the mortality at all ages, and one in less than 6 of the deaths occurring above fifteen years of age.

The waste of adult life from pulmonary consumption in England and Wales, that is to say, the number of deaths from consumption which might be prevented if all classes were placed in circumstances as favorable as those of the higher orders and of the professions, is estimated at 5000 a year, and a strong opinion is expressed by our author that this number is much below the truth. The estimate goes on the supposition that the waste from pulmonary consumption occurs only in the metropolis and about twenty of the largest cities, and that the only class whose lives are thus sacrificed is the labouring class. The great liability to pulmonary consumption of the class of tradesmen shows that a large addition might be made from this source.

We have already made a brief reference to the results of Dr. Guy's inspection of the workshops of the metropolis, especially of the printing-offices, in which, as is well known, there is a lamentable neglect of ventilation, and where gas-lights, and some one or other of those economists of heat, but not of life, stoves, steam-pipes, and hot-water tubes, add to the closeness and foulness of the air. In the results of these comparisons, we have a striking confirmation of the facts detailed in the first part of this abstract, and a proof of a position which experiments upon animals, the experience of the directors of the zoological gardens, and the general impression of all men who have given the subject a thought, firmly establishes—that a close, foul, heated atmosphere is a sure though slow poison, of which the fatal effects have been long shrouded from public view under the name of consumption, and the more surely concealed inasmuch as the popular belief attributes that fatal disease to some peculiarity of climate which England possesses in an unusual degree. Many other points of interest are examined in the papers before us by the aid of large numbers of facts, or more cursorily alluded to. For these, the reader must be referred to the essays of which the titles are given at the head of this abstract.

We shall conclude by allowing the author to portray, in his own words, the spirit in which he has conducted his arduous inquiries:

"In all investigations of this nature" he says, "there is much room for error. Some standards of comparison essential to accuracy, are at present wanting. Causes and effects are so mixed up that it is impossible to separate them. The disease which, by destroying the adult, puts a younger man into his place, also alters the distribution of the population, so as to swell the number of its own victims; and thus all attempts at perfect accuracy are rendered abortive. Approximations confessedly imperfect, and estimates necessarily rude, must hold the place of those accurate results which force conviction. The author would therefore again guard against misconception. He has not dared to characterize his results as certain or accurate, but merely as approximations to truth, and probabilities more or less strongly confirming one another. His estimates are open to correction, and await that correction at the hands of himself or others; but he trusts that in the absence of that certainty of which he is in search, the probabilities he has established will serve the purpose of attracting attention to a part of the great subject of public health which has hitherto received comparatively little attention, and in conclusion, he may be allowed to express his own conviction, that the evils which have been pointed out are not exaggerated."

---

